# Nutrient Limitation Inactivates Mrc1-to-Cds1 Checkpoint Signalling in *Schizosaccharomyces pombe*

**DOI:** 10.3390/cells7020015

**Published:** 2018-02-23

**Authors:** Jessica Fletcher, Liam Griffiths, Thomas Caspari

**Affiliations:** 1 School of Medical Sciences, Bangor University, Bangor LL57 2UW, UK; j.f.fletcher@swansea.ac.uk (J.F.); lbgriffiths90@gmail.com (L.G.); 2 Medical School, Swansea University, Swansea SA2 8PP, UK; 3 Postgraduate Doctoral Studies, Paracelsus Medical University, 5020 Salzburg, Austria

**Keywords:** replication, kinase, checkpoint, postmitotic, fission yeast, *S. pombe*, Cds1, starvation, glucose, nitrogen, cell cycle

## Abstract

The *S. pombe* checkpoint kinase, Cds1, protects the integrity of stalled DNA replication forks after its phosphorylation at threonine-11 by Rad3 (ATR). Modified Cds1 associates through its N-terminal forkhead-associated domain (FHA)-domain with Mrc1 (Claspin) at stalled forks. We report here that nutrient starvation results in post-translational changes to Cds1 and the loss of Mrc1. A drop in glucose after a down-shift from 3% to 0.1–0.3%, or when cells enter the stationary phase, triggers a sharp decline in Mrc1 and the accumulation of insoluble Cds1. Before this transition, Cds1 is transiently activated and phosphorylated by Rad3 when glucose levels fall. Because this coincides with the phosphorylation of histone 2AX at S129 by Rad3, an event that occurs towards the end of every unperturbed S phase, we suggest that a glucose limitation promotes the exit from the S phase. Since nitrogen starvation also depletes Mrc1 while Cds1 is post-translationally modified, we suggest that nutrient limitation is the general signal that promotes exit from S phase before it inactivates the Mrc1–Cds1 signalling component. Why Cds1 accumulates in resting cells while its activator Mrc1 declines is, as yet, unclear but suggests a novel function of Cds1 in non-replicating cells.

## 1. Introduction

Glucose limitation is a constant problem for cancer cells as they metabolise this sugar very rapidly to lactate by aerobic glycolysis in the cytoplasm [1,2,3]. The intermediates of aerobic glycolysis feed the anabolic pathways that provide protection from oxidative damage through the synthesis of NADPH [4]. High concentrations of lactate promote DNA repair, rendering cancer cells drug resistant [5]. Whether glucose limitation impacts also on the DNA damage checkpoints is currently unknown.

*Schizosaccharomyces pombe* provides an excellent opportunity to address this question since fission yeast cells resemble cancer cells in their ability to perform aerobic glycolysis (Warburg effect) [6]. Glucose starvation arrests proliferating *S. pombe* cells transiently in G2 through activation of the Cdc2 (CDK1) inhibitor, Wee1 kinase [7]. How Wee1 detects low glucose concentrations is unknown. Once glucose is exhausted, *S. pombe* cells react like cancer cells with an increase in oxidative stress [8,9]. This coincides with the phosphorylation of the MAP kinase, Sty1/Spc1 (p38), at threonine 171 and tyrosine 173 by the MAPK kinase, Wis1 [10]. Glucose availability is sensed by the cAMP-dependent kinase, Pka1, and it may be this pathway that stimulates Sty1/Spc1 prior to an increase in reactive oxygen [11,12,13]. The starvation signal may alternatively be generated by the cell integrity MAP kinase, PMK1 [14] or the phosphatase, Pyp1 [11,15]. The tyrosine phosphatase, Pyp1, together with its paralogue, Pyp2, dephosphorylates Sty1/Spc1 at Y173 [15]. Intriguingly, both phosphatases also regulate Wee1 [16,17]. Since Wee1 is essential for the starvation-induced G2 arrest [7], Pyp1 and Pyp2 may synchronise the cell cycle through the regulation of Sty1. 

The DNA damage checkpoint kinases, Cds1 (Chk2) and Chk1, phosphorylate Wee1 to block cell cycle progression in G2 when genotoxic stress is detected. Both kinases are activated by Rad3 (ATR) with only a minor role of the second checkpoint kinase, Tel1 (ATM). Rad3 phosphorylates Cds1 at threonine-11 to promote its association with the replication protein, Mrc1 (Claspin) at stalled DNA replication forks [18]. Chk1 is modified at serine-345 by Rad3 to arrest cell division in G2 when chromosomes are damaged [19]. 

Evidence for a possible link between glucose homoeostasis and the response to DNA damage has emerged so far from work with *Aspergillus nidulans*, *Saccharomyces cerevisiae* and human cells. In *Aspergillus*, the DNA damage sensor, ATM (Tel1), regulates mitochondrial activities and glucose uptake under starvation conditions [20], while in human cells, cytoplasmic ATM mobilises the intracellular glucose transporter pool in the response to insulin signalling [21]. In *S.cerevisiae*, ATR (Mec1) and Tel1 reduce the respiration rate of mitochondria in unchallenged cells and in the presence of DNA lesions, which suggests that checkpoint activation promotes aerobic glycolysis in the cytoplasm [22]. 

Our results reveal a novel link between glucose availability and Cds1 kinase. A sudden drop in glucose levels or entry into the stationary phase results in the loss of Mrc1 and the accumulation of insoluble and post-translationally changed Cds1 protein. These events occur after transient activation and phosphorylation of Cds1 by Rad3 when glucose levels fall during cell replication. Since the Rad3 dependent phosphorylation of histone 2AX at S129 coincides with the brief activation of Cds1, it is possible that cells try to exit DNA replication after a sudden withdrawal of glucose. We also show that nitrogen starvation, which initiates an exit from G1, results in similar changes to Mrc1 and Cds1. Taken together, this implies that nutrient limitation breaks up the Mrc1-Cds1 checkpoint unit when cells exit the cell cycle. 

## 2. Results

### 2.1. Glucose Starvation Transiently Activates Cds1

**Figure 1:**
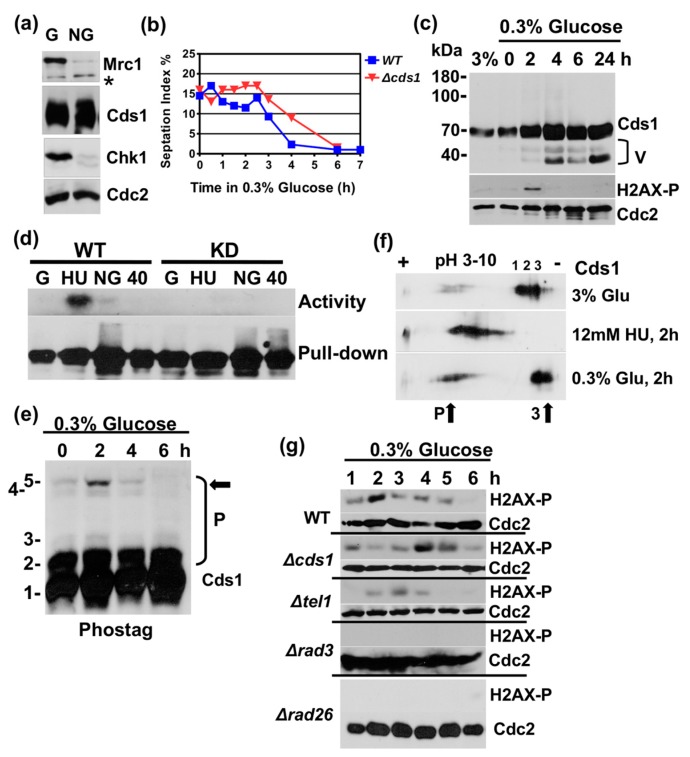
Glucose starvation transiently activates Cds1. (**a**) Cds1-HA_2_His_6_ (56 kDa), Chk1-HA_3_,(58 kDa), Mrc1 (180 kDa) and Cdc2 (35 kDa) in total protein extracts prepared from growing (G) and stationary cultures (NG) after 24 h of growth in rich medium with 3% glucose at 30 °C. (**b**) Septation index (G1/S cells) after the 3–0.3% glucose down-shift. (**c**) Cds1-HA_2_His_6_, Cdc2 and H2AX-S129-P in total protein extracts at 3% glucose and post-shift to 0.3% glucose. (**d**) In vitro kinase activity of immunoprecipitated Cds1-HA_2_His_6_ on myelin basic protein post-shift (top: phosphorylated myelin basic protein, bottom: precipitated Cds1 protein, asterisk: unspecific band). (**e**) Phos-tag SDS page of total protein extracts (P = phosphorylated forms 2–5, arrow = hyper-phosphorylated form 5). (**f**) Isoelectric focusing of Cds1-HA_2_His_6_ (P = more negatively charged species, arrows = changes in low glucose medium) (1–3) to more positively charged forms of Cds1 in 3% glucose medium. (**g**) H2AX-S129-P and Cdc2 in total protein extracts post-shift in wild type (WT) and selected deletion mutants.

We became interested in a link between Cds1 and glucose levels by a change observation. Fission yeast cells enter the stationary phase from G2 when glucose becomes limiting [10,23]. A Western blot analysis of total protein extracts from growing and stationary cultures revealed a sharp rise in Cds1 levels in post-mitotic cells while the amounts of Mrc1 and Chk1 both declined (Figure 1a). This was unexpected because Cds1 has so far only been associated with DNA replication in growing cells. 

To test whether Cds1 responds to changes in the glucose concentration, we shifted growing *cds1-HA_2_His_6_* cells [24] from 3% (166 mM) to 0.3% glucose in rich medium at 30 °C. The missing glucose molecules were replaced by 2.7% sorbitol, an inert carbon source, to avoid hypo-osmotic shock [25]. Cells continued to divide for approximately 150 min after the shift before the septation index started to drop (Figure 1b). The delayed decline in the septation index of cells without Cds1 (*Δcds1*) was not reproducible and was neither different from wild type, nor from cells lacking Rad3 or Tel1 (Figure S1a). We then analysed Cds1 protein levels, its in vitro kinase activity and phosphorylation patterns on a phos-tag SDS page and by isoelectric focusing (IF). While the phos-tag reagent slows down the mobility of phosphorylated proteins on the SDS page, IF detects any post-translational modifications that change the overall charge of the protein. 

Shortly before the septation index declined, around 2 h post-shift, two truncated bands of Cds1 accumulated (Figure 1c). Since the HA_2_His_6_ tag resides at the C-terminus, it is likely that both bands lack their N-termini. Informed by the presence of an internal translational start site in the *S. pombe rad9* checkpoint gene (M50) [26], we individually mutated the methionine codons in the *cds1* gene to alanine using the Cre-lox gene replacement method [27]. Only the mutation, M159A, that replaces the methionine residue between the forkhead-associated (FHA) and kinase domains (Figure S2c,d) prevented the appearance of the two inducible bands under glucose limitation conditions (Figure S2e). This strongly suggests that AUG-159 is utilized as an internal translational start site when glucose becomes limiting. It also implies that the second, larger band is a modified form of the inducible M159 variant. We were, however, not able to identify a biological function for this variant, as *cds1-M159A* mutant cells display no phenotype under starvation conditions. 

The in vitro kinase assay revealed a weak and transient activation of Cds1, approximately 2 h after the down-shift to 0.3% glucose that was abolished in the kinase-dead *cds1* mutant (D312E) (Figure 1d and Figure S1b). This activation was significantly lower compared to cells that were treated with the replication inhibitor, hydroxyurea, for 2 h (Figure 1d), suggesting that glucose limitation does not cause a strong DNA replication block. It was, however, intriguing to see that the phosphorylation of histone 2AX at S129 by Rad3 also transiently peaked 2 h post-shift (Figure 1c,g). Although this chromatin mark has been linked with DNA double strand breaks, it also increases during the unperturbed S phase [28]. Consistent with this report, we also detected a sharp rise in H2AX-S129 phosphorylation when synchronised cells were released back into the cell cycle from their mitotic *nda3.KM311* arrest. The *nda3.KM311* mutant gene encodes a cold-sensitive beta-tubulin protein that reversibly blocks spindle formation in mitosis [29]. Since our data show that the modification peak of H2AX coincides with the end of the S phase (Figure S2a,b), we concluded that a decline in glucose increases the number of forks that exit DNA replication. The weak activation of Cds1 may, therefore, indicate a role of the kinase in the coordination of the exit from S phase under nutrient starvation conditions. This conclusion is supported by the finding that the deletion of *cds1* (*Δcds1*) delayed the peak of H2AX-S129 phosphorylation by 2 h (Figure 1g). Hence, a loss of Cds1 may prolong the time replication forks need to terminate S phase when glucose becomes limiting. 

The results from the phos-tag assay were also in line with the transient activation of Cds1, 2 h post-shift, as Cds1 was highly phosphorylated at this time point (band 5 in Figure 1e). Consistent with these observations, the IF assay showed the appearance of two more negatively charged forms of Cds1, 2 h post-shift (Figure 1f, bottom panel). In addition to the phosphorylation of Cds1, the pattern of the three less negatively charged forms of Cds1, which were detected under normal glucose conditions (Figure 1f, No. 1, 2 and 3, top panel, 3% glucose), also changed when glucose was reduced. This suggests that further post-translational modifications affect Cds1, which are distinct from phosphorylation events. 

**Figure 2:**
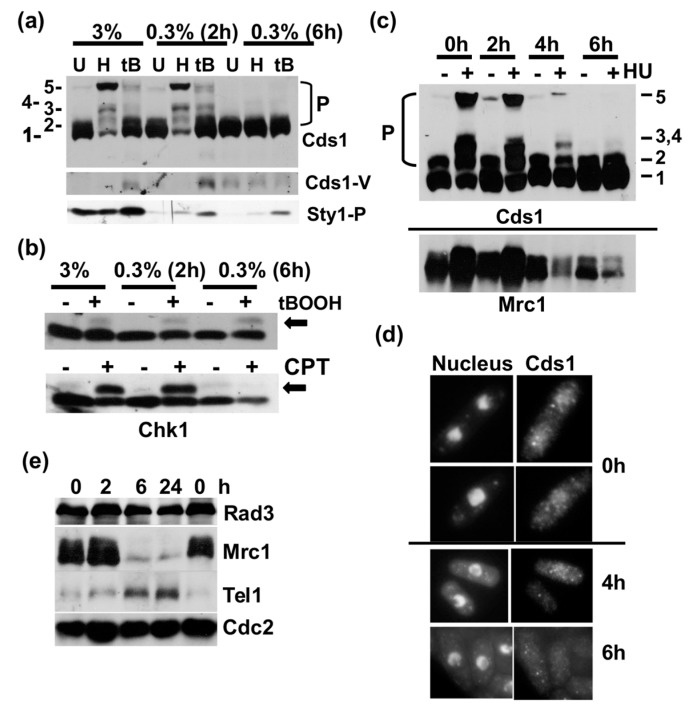
Low glucose concentrations terminate DNA replication. (**a**) Phos-tag SDS page of total protein extracts of Cds1-HA_2_His_6_ (U = untreated, H = 12 mM hydroxyurea (HU) for 3.5 h, tB = 0.5 mM t-butylhydroxyperoxide 3.5 h). HU and tB were added to growing cells (3%) at 2 h or 6 h post-shift to 0.3% glucose. Cds1-V = N-terminally truncated Cds1 variant, Sty1-P = dual T171 + Y173 phosphorylation. (**b**) SDS page of total protein extracts of Chk1-HA_3_; 0.5 mM tB and 12 μM camptothecin CPT were added at the indicated times and cells were incubated for another 2 h (arrow = Chk1-S345-P shift band). (**c**) Repeat experiment of (**a**). (**d**) Indirect immunofluorescence images of Cds1-Myc_13_ 0 h, 4 h and 6 h post-shift to 0.3% glucose (nucleus = DAPI, Cds1 = antibody signal). (**e**) SDS page of total protein extracts 0 h, 2 h, 6 h and 24 h post-shift to 0.3% glucose (Myc-Rad3, HA-Tel1).

Taken together, these findings support a model in which a decline in glucose results in the activation of Cds1 upon its phosphorylation by Rad3 and that this event is linked with the exit from DNA replication. This event is, however, different from a block of DNA replication by hydroxyurea (HU), as the latter very strongly activates Cds1 (Figure 1d) and causes a much more pronounced hyper-phosphorylation of the kinase as detected in the IF assay (Figure 1f, middle panel) and by phos-tag analysis (Figure 2a). The three less negatively charged forms of Cds1 (Figure 1f, No 1–3) all disappeared upon HU treatment giving way to a large number of more negatively charged, phosphorylated spots in the IF test.

### 2.2. Low Glucose Concentrations Terminate Cds1 Activation

DNA replication is an energy consuming process that requires a steady supply of glucose [30]. We used HU-induced activation of Cds1 via Mrc1 as an assay to find out when cells would stop DNA replication after the down-shift. Cds1-HA_2_His_6_ cells were shifted from 3% to 0.3% glucose and exposed to 12 mM HU or 0.5 mM t-butylhydroxyperoxide (tBOOH) at 2 h and 6 h after the reduction in this sugar. While HU stops DNA replication by depleting the nucleotide pool, tBOOH generates oxidative stress. The analysis of total protein extracts on the phos-tag SDS page revealed several highly phosphorylated bands of Cds1 (including band 5) after HU treatment in a normal medium (3%) and 2 h post-shift (Figure 2a). This strong modification of Cds1 is in line with the earlier IF experiment (Figure 1f, middle panel). tBOOH also activated Cds1, but to a lesser extent. Both chemicals lost, however, their stimulating activities 6 h post-shift, as Cds1 remained unmodified (Figure 2a). This result implies that asynchronous cells stopped replicating between 2 h and 6 h after the drop in glucose levels. We also monitored the dual phosphorylation of the MAP kinase, Sty1/Spc1, at T171 and Y173 in the same samples as a marker of oxidative stress. While tBOOH promoted the dual modification independently of the glucose concentration, we noticed a significant drop in the basal Sty1/Spc1 phosphorylation under glucose starvation conditions (Figure 2a, bottom panel, Figure S1c). Interestingly, the shorter Cds1 form was detectable after tBOOH treatment and in all samples 6 h post-shift (Figure 2a, middle panel). To test our conclusion that DNA replication stops 6 h post-shift, we analysed the Rad3-dependent phosphorylation of Chk1 at S345 (*chk1-HA_3_*) that is easily detected as a mobility shift [19]. This shift was present under all conditions after the addition of tBOOH but disappeared 6 h post-shift when DNA replication forks were damaged with the topoisomerase 1 inhibitor, camptothecin (CPT) (Figure 2b). CPT affects cells only in S phase, when replication forks collide with immobilised Top1-DNA complexes [31]. This confirms the absence of replicating cells 6 h after glucose withdrawal. We then repeated the experiment with the *cds1-HA_2_His_6_* strain to map, more closely, when cells cease S phase, and to analyse the amounts of Mrc1. Cds1 phosphorylation declined when HU was added 4 h post-shift, the same time as Mrc1 levels started to go down (Figure 2c). The loss of Mrc1 was more pronounced when cells were shifted from 3% to 0.1% glucose (Figure S1c) and was independent of Cds1 (Figure S1d). 

Since Cds1 monitors DNA replication in the nucleus [32], we tested whether low glucose affects its cellular localisation. Indirect immunofluorescence microscopy of Cds1-Myc_13_ cells showed an interesting decline in the nuclear antibody signal between 4 h and 6 h post-shift (Figure 2d). The loss of Cds1 from the nucleus could either indicate translocation to the cytoplasm or that the Myc-epitope becomes inaccessible inside the nucleus. Given the close link between Cds1 and its upstream kinases, Rad3 (ATR) and Tel1 (ATM) [18], we also analysed the abundance of both proteins in glucose-starved Myc-Rad3 and HA-Tel1 strains. While Rad3 levels remained largely unchanged, the amount of Tel1 was increased at 6 h and 24 h post-shift, coinciding with the disappearance of Mrc1 (Figure 2e). Tel1 is, however, not required for the decline in Mrc1 protein levels (Figure S1d). This data set supports a model in which a sudden drop in glucose suppresses DNA replication—an early event, which is then followed by a decline in Mrc1 levels and post-translational changes to Cds1 when cells exit the cell cycle from G2. 

### 2.3. Cds1 Undergoes a Transformation in the Stationary Phase

**Figure 3:**
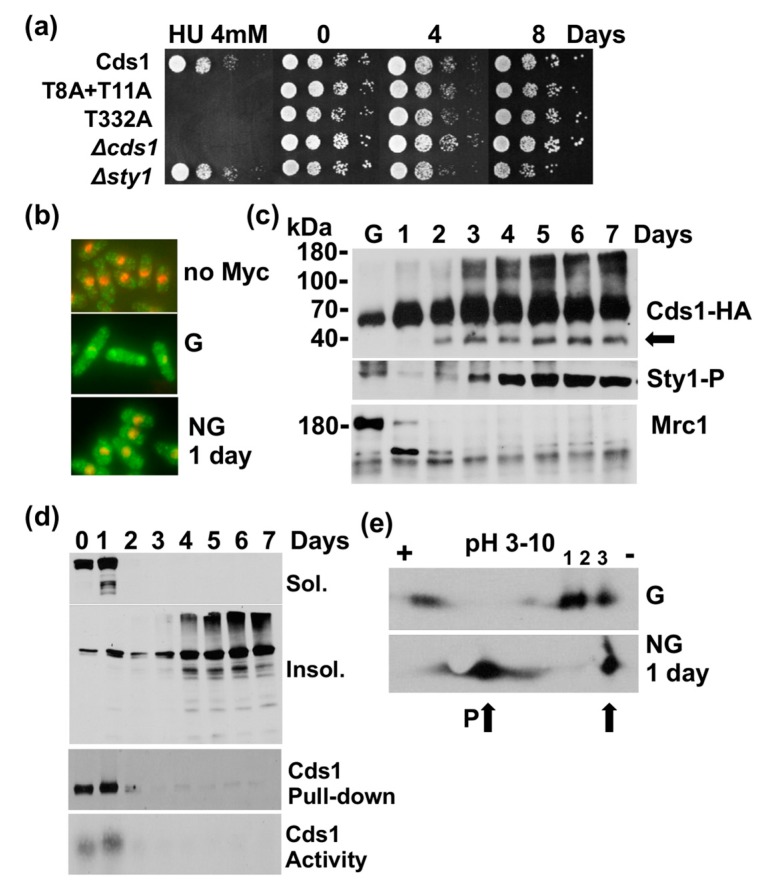
Cds1 undergoes a transformation in the stationary phase. (**a**) Survival of the indicated strains in 3% glucose medium. Samples were harvested on the indicated days in culture (stationary phase). Cell survival on 3% glucose in the presence of 4 mM hydroxyurea (HU) to verify the *cds1* strains. T8 and T11 are phosphorylated by Rad3; T332 is in the activation loop of the kinase domain of Cds1. (**b**) Indirect immunofluorescence images of Cds1-Myc_13_ in growing cells (G) and after 1 day in culture (stationary phase, NG) (overlay of red = DAPI and green = antibody signal). (**c**) Total protein extracts harvested at the indicated days in a 3% glucose culture (G = growing cells, arrow = N-terminally truncated Cds1 form, Sty1-P = dual T171 + Y173 phosphorylation, Mrc1 = 180 kDa band). Sty1-P and Mrc1 were analysed in the Cds1-HA_2_His_6_ extracts. (**d**) Samples were withdrawn on the indicated days in 3% glucose culture (sol. = soluble protein, insol. = Insoluble protein, immunoprecipitated Cds1-HA_2_His_6_ and in vitro kinase activity). (**e**) Isoelectric focusing of Cds1-HA_2_His_6_ (P = more negatively charged species. Arrows = changes in low glucose medium, G = growing cells, NG = 1 day in culture (stationary phase)).

To exclude the possibility that the changes affecting Cds1 are limited to the sudden removal of glucose, we followed Cds1-HA_2_His_6_ cells while they slowly metabolised this sugar during their transition from logarithmic growth into the stationary phase. Cells maintained their viability for up to 8 days in rich medium, and deletion of *cds1* had no impact on cell survival (Figure 3a). Consistent with the earlier tests, the nuclear Cds1 signal was lost after 1 day of growth (Figure 3b). After 2 days, the N-terminally truncated form appeared (Figure 3c, top panel), and the kinase became insoluble (Figure 3d). After 3 days, high molecular weight forms of Cds1 started to accumulate (Figure 3c, top panel). These findings support the earlier notion that a drop in glucose induces post-translational changes in Cds1, including the activation of the cryptic translational start site at M159 (Figure S2e). 

We also subjected total protein extracts of Cds1-HA_2_His_6_ cells to IF after 1 day of growth (stationary phase) and observed similar changes as after the sudden removal of glucose. One more negatively charged species of Cds1 appeared in stationary cells, while two of the three more positively charged forms were lost (Figure 3e, No 1 + 2). The drop in the nuclear signal and the changes in the post-translational modification pattern correlated with a steep decline in Mrc1 levels after 1 day of growth (Figure 3c, bottom panel). The dual phosphorylation of Sty1/Spc1 dropped initially as after the acute removal of glucose but started to accumulate again on the third day in the stationary phase (Figure 3c, middle panel). 

These results confirm the earlier findings and strongly suggest that Cds1 undergoes significant changes when cells exit the cell cycle in the response to glucose starvation. 

### 2.4. Nitrogen Starvation Causes Similar Changes to Cds1 and Mrc1

**Figure 4:**
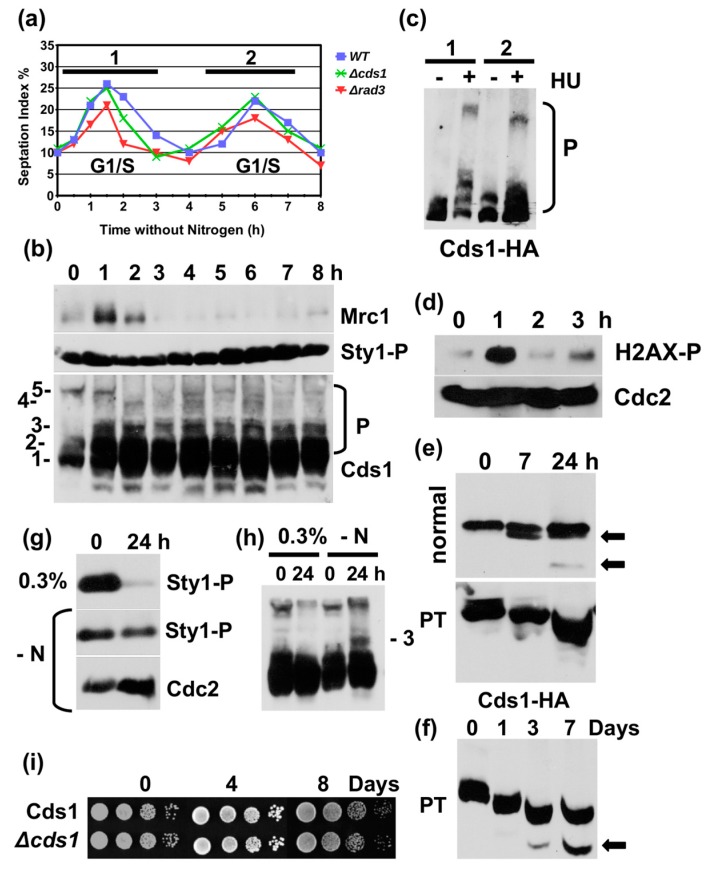
Nitrogen starvation causes similar changes to Cds1 and Mrc1. (**a**) Cds1-HA_2_His_6_, cds1 deletion and rad3 deletion strains without auxotrophic markers were grown in minimal medium with nitrogen overnight, washed and resuspended in minimal medium without nitrogen at 30 °C. The rise in the septation index (G1/S cells) shows the two rounds of DNA replication post-shift. (**b**) Total protein extracts tested at the indicated hours post-shift and probed for Mrc1, Sty1-T171P + Y173P and Cds1-HA_2_His_6_, (P = phosphorylated Cds1 protein on phostag SDS page), (**c**) Phostag SDS page of Cds1-HA_2_His_6_. Twelve mM HU was added at 20 min (1) post-shift or 4 h (2) post-shift. Cells were harvested 2 h later. (**d**) Total protein extracts analysed at the indicated hours post-shift and probed for H2AX-S129-P and Cdc2. (**e**) Normal and phostag (PT) SDS page of Cds1-HA_2_His_6_, harvested 0 h, 7 h and 24 post-shift. (**f**) PT-SDS page of Cds1-HA_2_His_6_ cells, harvested at 0 h, 1 h, 3 h and 7 h (arrow = N-terminally truncated Cds1 form). (**g**) SDS page of Sty1-T171P+Y173P in 0.3% glucose or in minimal medium without nitrogen (-N) at 0 h and 24 h. (**h**) PT-SDS page of Cds1-HA_2_His_6_ in 0.3% glucose or in minimal medium without nitrogen (-N) at 0 h and 24 h (3 = phosphorylated form). (**i**) Survival of the indicated strains in minimal medium without nitrogen.

Although our data are consistent with a role of glucose in the regulation of Cds1, we could not rule out that the latter events are an indirect outcome of a starvation-induced cell cycle exit. Since fission yeast cells enter the stationary phase from G2 after glucose withdrawal [23], we also investigated Cds1 in nitrogen starved cells, as they exit from G1 to allow the onset of sexual differentiation [33]. After the removal of all auxotrophic markers from the *cds1-HA_2_His_6_* strain, cells were first grown overnight at 30 °C in minimal medium with nitrogen and then resuspended in the medium without this nutrient. As reported previously [34], cells underwent two consecutive cell cycle rounds within 8 h after the shift (Figure 4a). Soon after the removal of nitrogen (1 h post-shift), we noticed the shorter Cds1 variant and a strong increase in phospho-band number 3 of Cds1 in the total protein extracts. Interestingly, the phospho-band 3 appeared to be specific to nitrogen starvation (Figure 4h). Both changes remained present throughout the time course (Figure 4b). While Mrc1 levels peaked during the first cell division, 1 h post-shift (Figure 4b, top panel), as did the S129 phosphorylation of histone 2AX (Figure 4d), the amounts of Mrc1 stayed very low during the second cycle (Figure 4b, top panel). This decline could be because the last (second) S phase before meiosis differs from a normal mitotic round of DNA replication [35]. In contrast to glucose starvation, the MAP kinase, Sty1/Spc1, was highly phosphorylated at T171 and Y173 throughout the time course (Figure 4b,g). This high level of MAP kinase activation is typical for cells grown in minimal medium [36]. When we tested the responsiveness of Mrc1-to-Cds1 signalling by adding 12 mM HU, either before the first cycle (20 min after nitrogen removal) or prior to the second round (4 h after nitrogen removal), Cds1 phosphorylation was clearly reduced in total protein extracts during the second cycle when Mrc1 levels were low (Figure 4c). We then analysed soluble protein extracts of *cds1-HA_2_His_6_* cells on an SDS page with and without phos-tag. Consistent with the earlier findings, we detected a reduction in Cds1 phosphorylation at the time of the second cycle. Seven hours post-shift, the Cds1 band became a double band, an event that coincided with the loss of a slower migrating, phosphorylated band in the presence of the phos-tag agent (Figure 4e). This subtle dephosphorylation continued for the next three days in minimal medium without nitrogen (Figure 4e,f). The truncated Cds1 band appeared 24 h after the removal of nitrogen in the soluble extract and continued to accumulate in resting cells (Figure 4e,f). Cells without Cds1 maintained viability throughout the experiment (Figure 4i). 

In summary, these findings argue that most key changes to Cds1 and Mrc1 are a more general response to nutrient limitation and the subsequent exit from the cell cycle. Why Cds1 accumulates in post-mitotic cells, while Mrc1 disappears, remains to be understood, since cells without Cds1 do not lose viability under starvation conditions. The cellular functions of the inducible, N-terminal truncated form of Cds1 are also still enigmatic as the Cds1-M159A strain displays no starvation-related phenotypes. Although nutrient limitation or the resulting cell cycle exit appear to be the general signal, there were some differences between glucose and nitrogen starvation. The high molecular weight forms of the kinase accumulated only under glucose limitation conditions, whereas phospho-band 3 appeared only in the absence of nitrogen.

## 3. Discussion

This work identified a novel link between nutrient availability and the termination of Mrc1-to-Cds1 signalling. Glucose starvation initially triggers the transient phosphorylation and activation of Cds1 that coincides with the modification of histone 2AX at S129 by Rad3 2h after the down-shift (Figure 1). Since Cds1 is phosphorylated at stalled DNA replication forks and H2AX towards the end of S phase [28,37] (Figure S2b), it is quite possible that a glucose shortage increases the number of replication forks that exit S phase. Such a synchronising effect was observed in bacteria, where DNA replication stops when nutrients become limiting [38] and in *S. cerevisiae*, where fork elongation slows down as a starvation response [39]. Prolonged starvation of *S. pombe* cells leads to the removal of Mrc1 (Figure 2c,e and Figure S1c,d), the protein that couples Cds1 to stalled DNA replication forks. This transition occurs between 4 h and 6 h post-shift when DNA replication stops (Figure 2c). The nuclear signal of Cds1 also declines during this time (Figure 2d) suggesting that the kinase either moves to the cytoplasm or undergoes modifications inside the nucleus. Once cells enter the stationary phase, no nuclear Cds1 signal is detectable (Figure 3b), and high molecular weight forms of Cds1 accumulate (Figure 3c). Why stationary cells acquire large amounts of Cds1 in the absence of its activator Mrc1 remains to be explained. Cds1 is not important for viability (Figure 3a) and not required for the induction of the glucose transporters, Ght4 and Ght5 (Figure S3). We tested both transport proteins, as their expression is induced when cells enter the stationary phase [40]. 

How a glucose shortage initiates this transition is not yet clear. This could be an indirect consequence of an exit from DNA replication which then triggers a breakdown of Mrc1 when cells arrest in G2. Mrc1 is a labile protein that oscillates during the cycle with a peak in S phase [41]. The changes in the post-translational modification pattern of Cds1 2 h post shift (Figure 1f) and in the stationary phase (Figure 3e), which are distinct from its Mrc1-dependent modification at stalled forks, support a model in which Cds1 becomes a target of a kinase. The AMP-activated protein kinase, Snf1/Ssp2, is a strong candidate for such a regulator. The subunit, Snf4, of this key glucose sensor is genetically linked with Cds1 (Chk2) during eye development in *Drosophila* [42]. Intriguingly, *S. pombe* Snf1/Ssp2 interacts physically with the replication ATPase, Mgt1 [43] ,which could provide a link between the exit from DNA replication and glucose sensing. Hence, an exit from DNA replication in the response to nutrient limitation could activate Snf1/Ssp2 kinase which, subsequently, phosphorylates Cds1. It should, however, also be noted that such a regulation could go through a phosphatase as a second Cds1 pool becomes more positively charged, which indicates a loss of negatively charged phosphate groups (Figure 1f and Figure 3e). 

From the group of DNA damage checkpoint proteins, only ATM (Tel1) and ATR (Rad3, Mec1) have, so far, been linked with the regulation of glucose homoeostasis. Human ATM locates to the cytoplasm to activate the glucose transport proteins, GLUT1 and GLUT4 [21,44]. *Aspergillus* ATM regulates mitochondrial functions and glucose uptake under starvation conditions [20]. In *S. cerevisiae*, ATM (Tel1) and ATR (Mec1) modulate the activity of Snf1/Ssp2 kinase indirectly via the SUMO E3 ligase Mms21, to control glucose fermentation [22]. Mec1 (ATR) is also phosphorylated by Snf1/Ssp2 on the mitochondrial surface to recruit Atg1 which ensures mitochondrial respiration during glucose starvation [45]. Taken together, these findings are in line with our hypothesis that the AMP-activated protein kinase, Snf1/Ssp2, is a strong candidate as the regulator of Cds1 under glucose limiting conditions. It should also be noted that the protein levels of Tel1 (ATM) increase after a sudden drop in glucose availability (Figure 2e).

The changes to Cds1 are not specific to glucose scarcity as nitrogen starvation induces similar events. One pool of Cds1 becomes phosphorylated (band 3 in Figure 4b,h), while a second pool, the soluble kinase, is dephosphorylated (Figure 4e,f). The phospho-form number 3 appears to be unique to nitrogen limitation (Figure 4h) and the amount of insoluble kinase is also lower than under glucose limitation conditions. Also Mrc1 levels drop after extended nitrogen shortage as they do when glucose becomes scarce. The two Target of Rapamycin (TOR) kinases, Tor1 and Tor2, are good candidates for the regulation of Cds1 under nitrogen limitation and perhaps also under glucose starvation conditions, as they are regulated by the availability of both nutrients [46,47] and converge on the same down-stream targets [48]. 

In summary, our results support a novel, but yet unknown, role of the DNA replication checkpoint kinase, Cds1, in post-mitotic cells under nutrient limiting conditions. Further work is, however, required to identify this role and to dissect how nutrient limitation terminates Mrc1-to-Cds1 signalling.

## 4. Materials and Methods

### 4.1. Yeast Strains

The genotype of the strains used in this study was *ade6-M210 leu1-32 ura4-D18*. The *rad3* gene was deleted with the *ade6+* gene, the genes *cds1*, *sty1* and *rad26* gene were deleted with *ura4+* and the *tel1* gene was deleted with *leu2+*. The following strains were used: cds1-HA_2_His_6_∷ura4+, cds1-T8A+T11A-HA_2_His_6_, cds1-T332A-HA_2_His_6_ [18], cds1-M159A-HA_2_His_6_ (this study), *cds1-D312E-HA_2_His_6_*, *cds1-Myc_13_* [49] *chk1-HA_3_* [50], Myc-Rad3 and HA-Tel1 [51], Dfp1-His_6_HA_3_ *nda3.KM311* [52], Ght4-GFP and Ght5-GFP [40].

### 4.2. Media

The glucose experiments were performed in yeast extract-adenine (YEA) medium (3% glucose, 0.5% yeast extract, 100 mg/L adenine) at 30 °C. The shift medium contained 0.1–0.3% glucose, 2.9–2.7% sorbitol, 0.5% yeast extract and 100 mg/L adenine. Nitrogen starvation experiments were performed in minimal medium (3% glucose, 0.67% yeast nitrogen base with or without ammonium sulphate (Formedium)) at 30 °C.

### 4.3. Phos-tag SDS Page

Phos-tag gels (6%) were prepared and run as reported in [53]. The gel was prepared with phos-tag acrylamide (AAL-107, Wako Ltd., Hong Kong, China). 

### 4.4. Isoelectric Focusing (IF)

IF experiments were performed on linear strips of pH 3–10 (Biorad, Hercules, CA, USA, rapid ΔV), as described in [53]. The strips used were IEF Dry Strips, pH 3–10, linear (Biorad, 163–2000).

### 4.5. In Vitro Kinase Assay

Cds1 activity was measured as performed in [37] using anti-HA (haemagglutinin) magnetic beads (Pierce, 88836).

### 4.6. Survival Assays

The drop tests assays used are described in [54].

### 4.7. Indirect Immunofluorescence Microscopy 

Cds1-Myc_13_ was detected as reported in [55].

### 4.8. Cell Synchronisation

The *nda3.KM311* mitotic arrest was performed in rich medium as reported in [29]. S phase is indicated by the phosphorylation peak of Dfp1 [52].

### 4.9. Antibodies

The antibodies used were as follows: anti-HA antibody (BioScource, Covance MMS-101P-200), anti-Mrc1 antibody (ABCAM, ab188269), anti-Cdc2 antibody (ab5467), anti-Sty1/p38 T171-P + Y173-P (ABCAM ab4822), anti-histone 2AX-S129-P antibody (ab17576), anti-Rad4/Cut5 antibody (ab79775), anti-Myc antibody (Santa Cruz SC-40), secondary mouse-HRP (Dako, Beijing, China, P0447), secondary rabbit-HRP (Dako, P0217) and secondary-goat anti-mouse-Alexa Fluor 488 (Molecular Probe A-11029).

## Supplementary Materials

The following are available online at http://www.mdpi.com/2073-4409/7/2/15/s1.
