# Supplementary material for: Nutrient Limitation Inactivates Mrc1-to-Cds1 Checkpoint Signalling in Schizosaccharomyces pombe
Source: Cells. 2018 Feb 23;7(2):15. doi: 10.3390/cells7020015 (PMC5850103; doi:10.3390/cells7020015)
Supplement: Supplementary file 1 [file cells-07-00015-s001.pdf]

Supplementary Materials for

Nutrient limitation inactivates Mrc1-to-Cds1 checkpoint signalling in *Schizosaccharomyces pombe*

Jessica Fletcher[1,2], Liam Griffiths[1] and Thomas Caspari[1,3]*

1: Bangor University, School of Medical Sciences, Bangor LL57 2UW, United Kingdom, email: t.caspari@bangor.ac.uk, orcid ID: 0000-0002-1450-4774, email Liam Griffiths: lbgriffiths90@gmail.com

2: Present address: Swansea University, Medical School, Swansea, SA2 8PP, United Kingdom, email: j.f.fletcher@swansea.ac.uk orcid ID: 0000-0002-4911-2711

3: Present address: Paracelsus Medical University, Postgraduate Doctoral Studies, 5020 Salzburg, Austria, email: thomas.caspari@pmu.ac.at phone: 0043-662-2420-80245

* Corresponding Author. email: thomas.caspari@pmu.ac.at phone: 0043-662-2420-80245

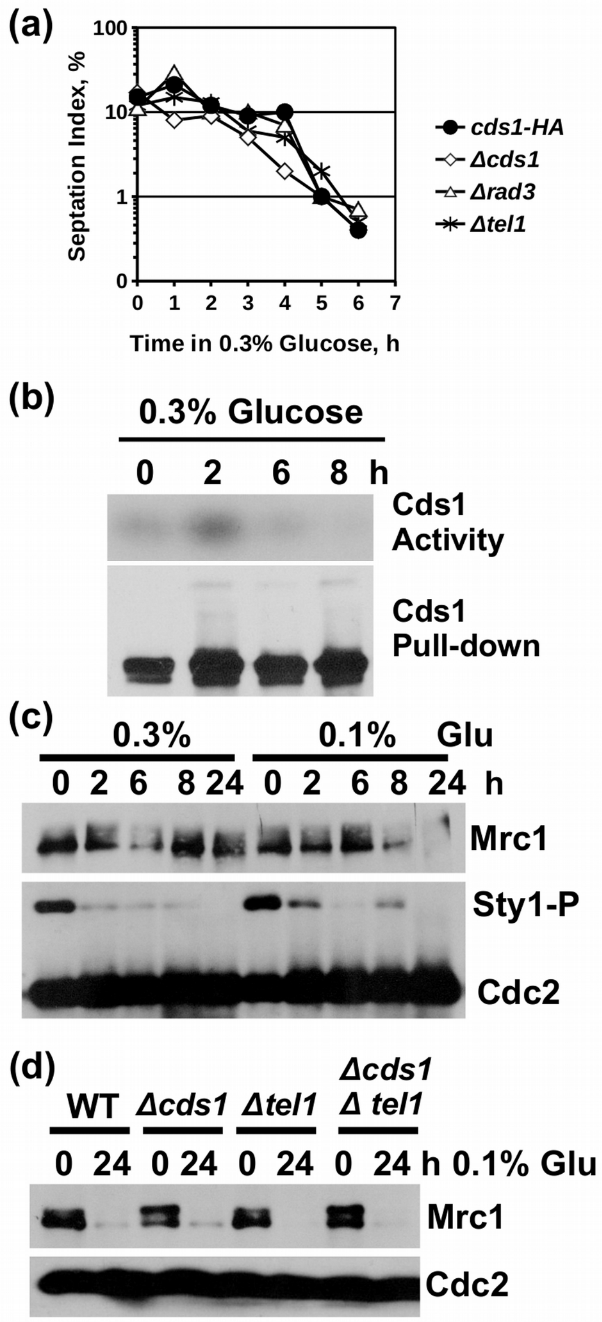

Supplementary Figure 1. Glucose starvation transiently activates Cds1 and removes Mrc1. (a) Cds1-HA_2His_6, *Δcds1*, *Δrad3* and *Δtel1* cells were grown in 0.3% glucose rich medium at 30°C for the indicated times. Samples were withdrawn, fixed in methanol and scored for septated cells after staining the DNA with DAPI and the new septum with calcofluor (septation index = cells in G1/S). (b) Cds1-HA_2His_6 (56kDa) protein was immunoprecipitated at the indicated times and used to phosphorylate myelin basic protein *in vitro* after a shift from 3% to 0.3% glucose in rich medium at 30°C. (c) Total protein extracts prepared at the indicated times after a down-shift to 0.3% or 0.1% glucose in rich

medium at 30°C. Probed for Mrc1, Sty1-T171P+Y173P and Cdc2. (d) Total protein extracts prepared at 0h and 24h after the down-shift to 0.1% glucose in rich medium at 30°C. Probed for Mrc1 and Cdc2 in wild type cells (WT) and cells deleted for the indicated genes.

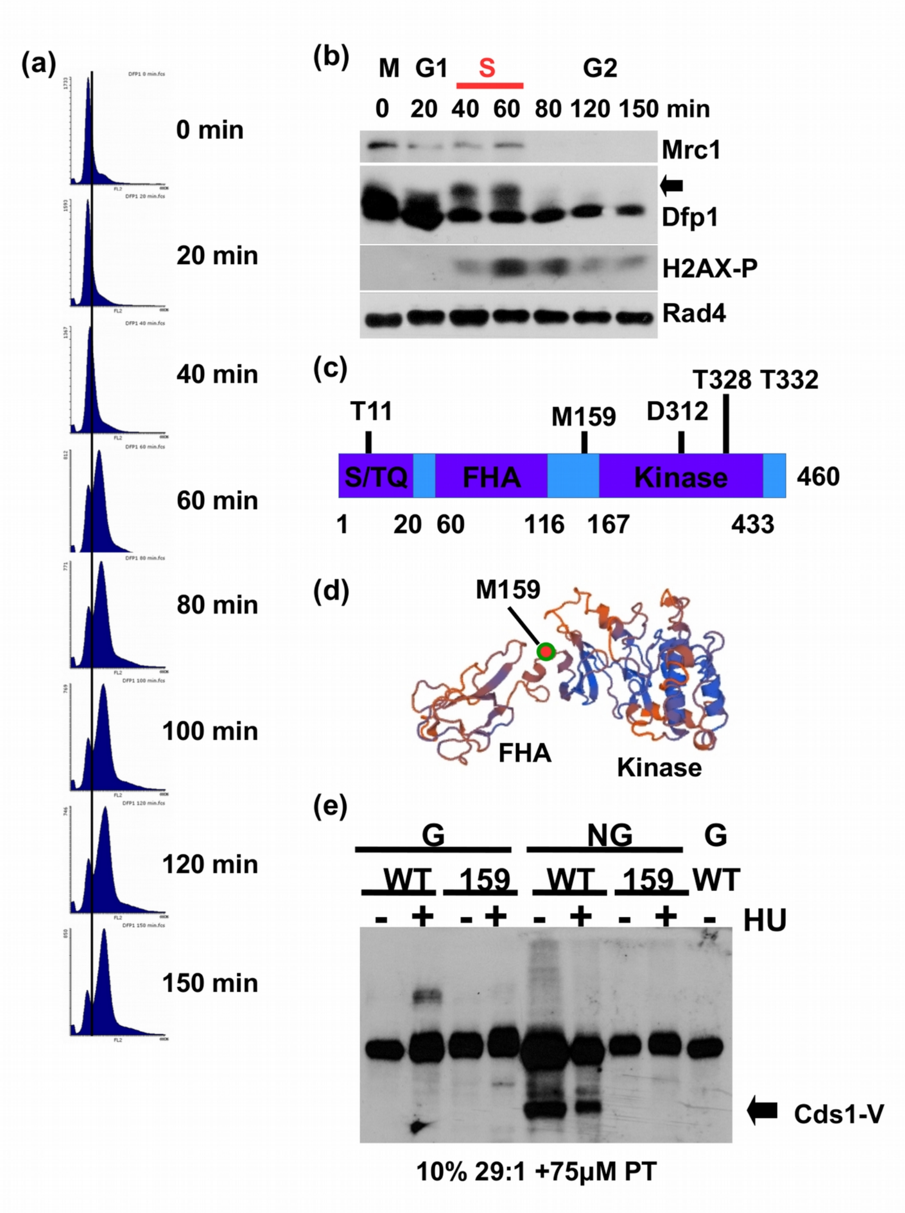

Supplementary Figure 2. Histone 2AX is phosphorylated towards the end of S phase at serine 129 and M159 is a cryptic translational start codon in the *cds1* mRNA.

(a) *Dfp1-His$_6$HA$_3$ nda3.KM311* cells were synchronised in mitosis at 20°C for 8h in rich medium with 3% glucose and then quickly released into rich medium with 3% glucose at 30°C. The DNA content was measured using a CUBE 8 (Sysmex) instrument and the histograms were produced using the free Flowing Software (http://flowingsoftware.btk.fi/). Cells completed S phase between 40 min and 60 min post-release. (b) Total protein extracts were probed for Dfp1-His$_6$HA$_3$ with an anti-HA antibody and for Mrc1, H2AX-S129-P and Rad4 with the corresponding antibodies. Dfp1 is hyper-modified as indicated by the

upper shift band in S phase (arrow). H2AX phosphorylation by Rad3 peaks towards the end of S phase when both, Dfp1 modification and Mrc1 are down-regulated. (c + d) Location of the cryptic translational initiation codon AUG-159 (methionine 159) between the N-terminal FHA and C-terminal kinase domain of Cds1. The model of Cds1 is based on human Chk2 kinase (PDB ID: 3I6U) The model was produced with the Swiss Modelling tool (https://swissmodel.expasy.org/) . (e) Total protein extracts of Cds1-HA_2His_6 and Cds1-M159A-HA_2His_6, cells were prepared from growing (G) and non-growing (NG) (24h growth in 3% glucose rich medium at 30°C) cells. Cells were incubated with 12mM HU for 2h (+HU) under both conditions. Cds1 is only hyper-phosphorylated in growing cells, while its N-terminally truncated band is limited to non-growing, starved cells. The latter band is absent when M159 is replaced by an alanine codon (M159A). The second band above the shorter form may be a phosphorylated variant of the latter (arrow). PT = phostag SDS page.

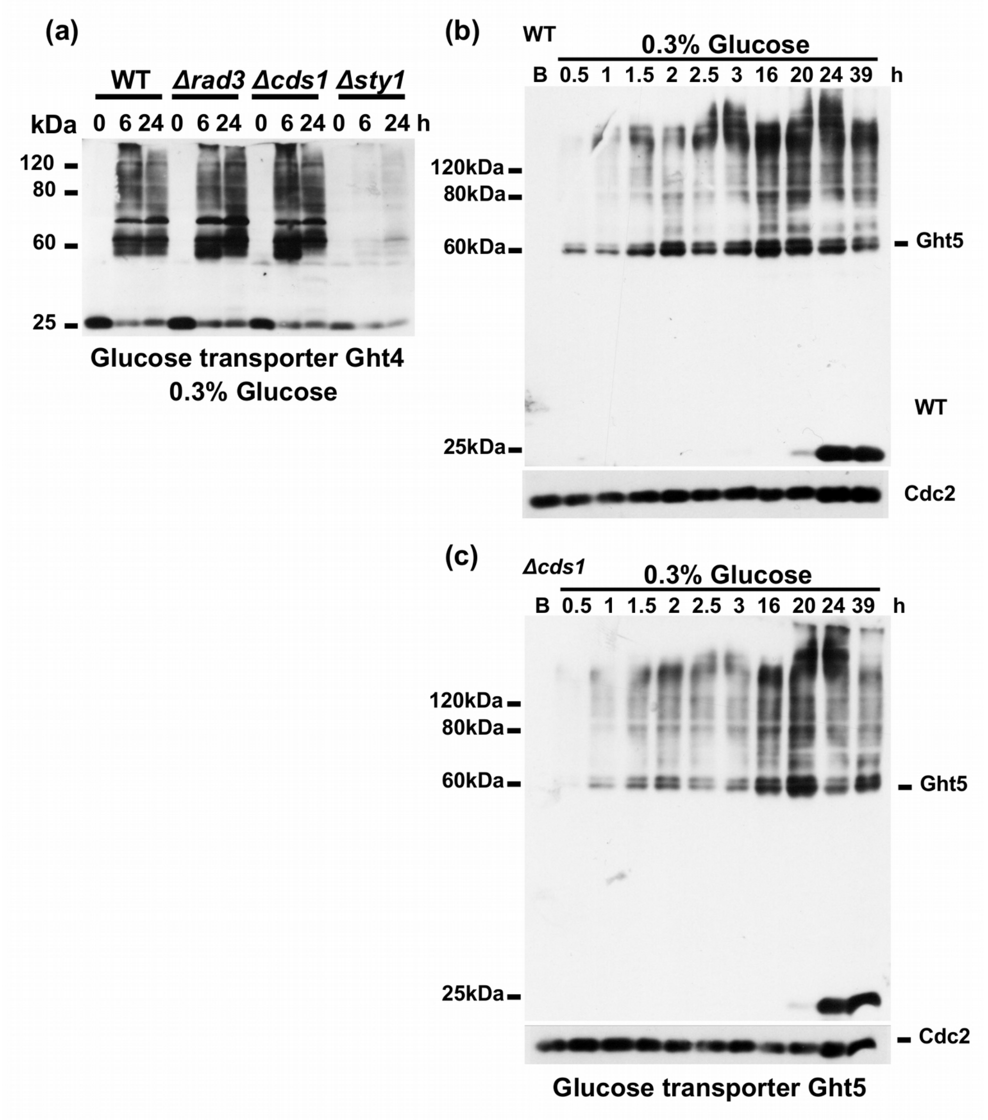

Supplementary Figure 3. Cds1 is not required for the induction of the glucose transporter Ght4 and Ght5. (a) Ght4-GFP wild type (WT) cells or Ght4-GFP strains deleted for the indicated genes were shifted to 0.3% glucose and total protein extracts were prepared at the indicated times. The molecular weight is ~65kDa. Highly modified forms are detectable after induction. Up-regulation requires the MAP kinase Sty1/Spc1. (b) Induction of Ght5-GFP in wild type cells. The molecular weight is ~65kDa. A 25kDa breakdown product is detectable after 20h in 0.3% glucose medium. (c) Same experiment in a *cds1* deletion background.
